# Supplementary material for: Rapid Extraction of Viral Nucleic Acids Using Rotating Blade Lysis and Magnetic Beads
Source: Diagnostics (Basel). 2022 Aug 17;12(8):1995. doi: 10.3390/diagnostics12081995 (PMC9407373; doi:10.3390/diagnostics12081995)
Supplement: Supplementary file 1 [file diagnostics-12-01995-s001.zip › diagnostics-1822913-supplementary.pdf]

diagnostics

MDPI

Article

Rapid Extraction of Viral Nucleic Acids Using Rotating Blade Lysis and Magnetic Beads

Minju Bae [1], Junsoo Park [2], Hyeonah Seong [1], Hansol Lee [3], Wonsuk Choi [4], Jiyun Noh [4], Woojoo Kim [3,4,*] and Sehyun Shin [1,2,5,*]

1 School of Mechanical Engineering, Korea University, Seoul 02841, Korea
2 Department of Micro-Nano Systems, Korea University, Seoul 02841, Korea
3 Asia Pacific Influenza Institute, Korea University College of Medicine, Seoul 02841, Korea
4 Division of Infectious Diseases, Department of Internal Medicine, Korea University College of Medicine, Seoul 02841, Korea
5 Engineering Research Center for Biofluid Biopsy, Seoul 02841, Korea
* Correspondence: wjkim@korea.ac.kr (W.J.K.); lexerdshin@korea.ac.kr (S.S.); Tel.: +82-2-2626-3051 (W.J.K.); +82-2-3290-3377 (S.S.)

* To whom correspondence should be addressed:

Sehyun Shin

Telephone: (82) 2-3290-3377

E-mail: lexerdshin@korea.ac.kr

Table S1. Clinical characteristics and sequence analysis of patients with influenza A virus infections.

Sample	Hospital	Sex	Age	Sampling Date	RAT	PCR (Ct Value)	
						A/H1	A/H3
D1816	Ansan, Korea University	M	56	2020-01-14	FluA	-	23.215832
A2911	Guro, Korea University	F	39	2020-01-06	FluA	24.40932	-
A2887	Guro, Korea University	F	49	2019-12-26	FluA	20.92608	-

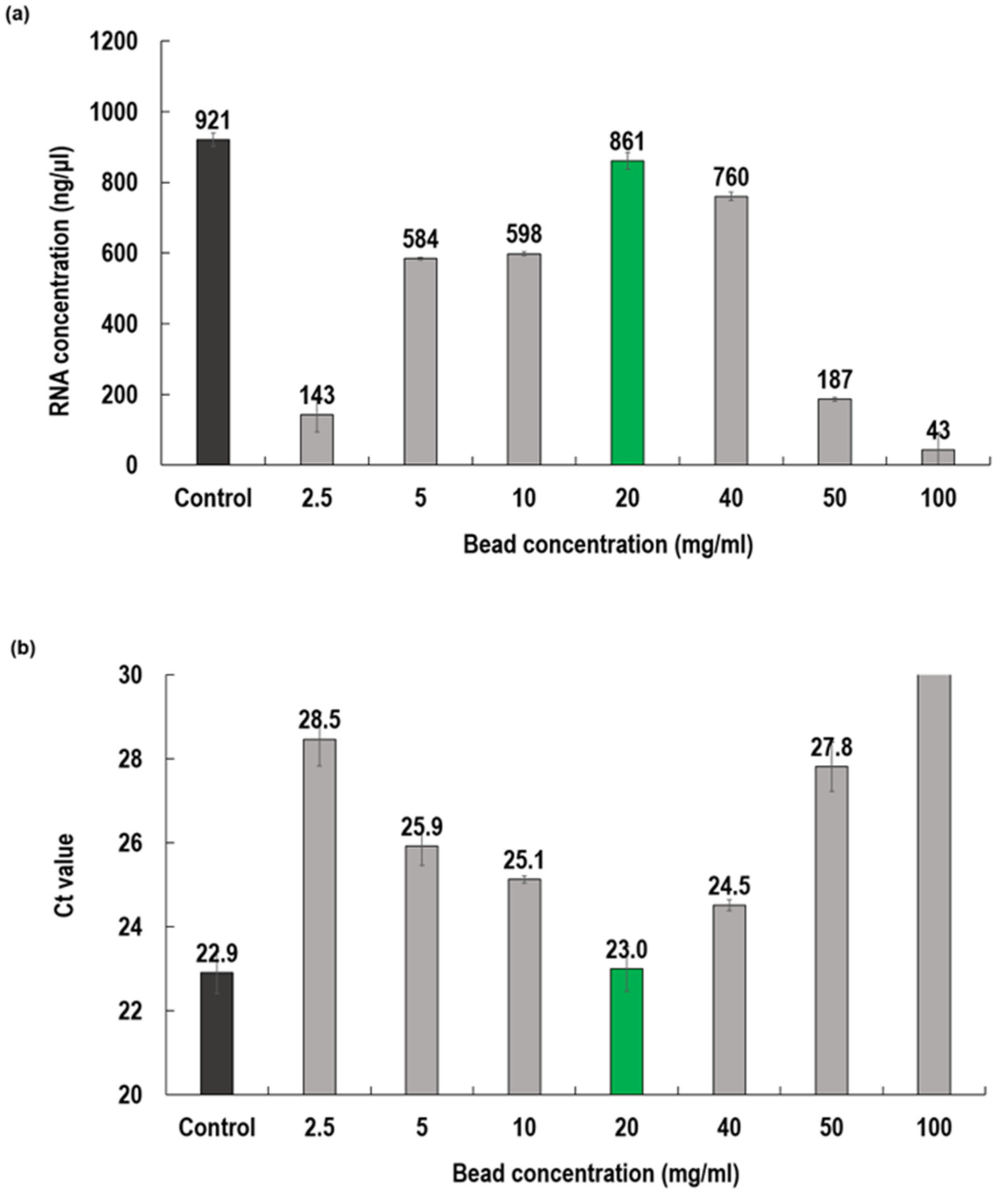

Figure S1. Effect of magnetic bead concentration on viral RNA extraction compared to control. (**a**) Extracted RNA concentrations, (**b**) Threshold cycles of PCR. Control:QIAamp (Qiagen).
